# Early-Outcome Differences between Acute and Chronic Periprosthetic Joint Infections—A Retrospective Single-Center Study

**DOI:** 10.3390/antibiotics13030198

**Published:** 2024-02-20

**Authors:** Yasmin Youssef, Elisabeth Roschke, Nadine Dietze, Anna-Judith Dahse, Iris F. Chaberny, Donald Ranft, Christina Pempe, Szymon Goralski, Mohamed Ghanem, Regine Kluge, Christoph Lübbert, Arne C. Rodloff, Andreas Roth

**Affiliations:** 1 Department of Orthopedics, Traumatology and Plastic Surgery, University Hospital Leipzig, Liebigstraße 20, 04103 Leipzig, Germany; elisabeth.roschke@medizin.uni-leipzig.de (E.R.); christina.pempe@medizin.uni-leipzig.de (C.P.); szymon.goralski@medizin.uni-leipzig.de (S.G.); mohamed.ghanem@medizin.uni-leipzig.de (M.G.); andreas.roth@medizin.uni-leipzig.de (A.R.); 2 Institute of Medical Microbiology and Virology, University Hospital Leipzig, Liebigstraße 21, 04103 Leipzig, Germany; nadine.dietze@medizin.uni-leipzig.de (N.D.); acr@dg-email.de (A.C.R.); 3 Hospital Pharmacy, University Hospital Leipzig, Liebigstraße 20, 04103 Leipzig, Germany; anna-judith.dahse@medizin.uni-leipzig.de (A.-J.D.); donald.ranft@medizin.uni-leipzig.de (D.R.); 4 Institute of Hygiene, Hospital Epidemiology and Environmental Health, University Hospital Leipzig, Liebigstraße 22, 04103 Leipzig, Germany; iris.chaberny@medizin.uni-leipzig.de; 5 Institute of Hospital Epidemiology and Environmental Hygiene, University Medical Center Schleswig-Holstein, Arnold-Heller-Str. 3, 24105 Kiel, Germany; 6 Department of Nuclear Medicine, University Hospital Leipzig, Liebigstraße 18, 04103 Leipzig, Germany; regine.kluge@medizin.uni-leipzig.de; 7 Division of Infectious Diseases and Tropical Medicine, Department of Medicine I, University Hospital Leipzig, Liebigstraße 20, 04103 Leipzig, Germany; christoph.luebbert@medizin.uni-leipzig.de; 8 Interdisciplinary Center for Infectious Diseases, University Hospital Leipzig, Liebigstraße 20, 04103 Leipzig, Germany

**Keywords:** periprosthetic joint infection, arthroplasty, outcome, infectious focus, complication

## Abstract

Periprosthetic joint infections (PJI) are serious complications after arthroplasty, associated with high morbidity, mortality, and complex treatment processes. The outcomes of different PJI entities are largely unknown. The aim of this study was to access the early outcomes of different PJI entities. A retrospective, single-center study was conducted. The characteristics and outcomes of patients with PJI treated between 2018 and 2019 were evaluated 12 months after the completion of treatment. Primary endpoints were mortality, relapse free survival (RFS) and postoperative complications (kidney failure, sepsis, admission to ICU). A total of 115 cases were included [19.1% early (EI), 33.0% acute late (ALI), and 47.8% chronic infections (CI)]. Patients with ALI were older (*p* = 0.023), had higher ASA scores (*p* = 0.031), preoperative CRP concentrations (*p* = 0.011), incidence of kidney failure (*p* = 0.002) and sepsis (*p* = 0.026). They also tended towards higher in-house mortality (ALI 21.1%, 13.6% EI, 5.5% CI) and admission to ICU (ALI 50.0%, 22.7% EI, 30.9% CI). At 12 months, 15.4% of patients with EI had a relapse, compared to 38.1% in ALI and 36.4% in CI. There are differences in patient characteristics and early outcomes between PJI entities. Patients with EI have better early clinical outcomes. Patients with ALI require special attention during follow-up because they have higher occurrences of relapses and postoperative complications than patients with EI and CI.

## 1. Introduction

Periprosthetic joint infections (PJIs) are serious and challenging complications following total joint arthroplasty and have a significant effect on overall clinical outcomes and patient satisfaction. They are associated with higher morbidity and mortality, require more complex treatment strategies, and lead to increased healthcare costs [1,2,3]. According to the 2019 German Endoprosthesis Registry Report, PJIs are the second most common cause of revision surgery after total hip and knee arthroplasty in Germany [4].

PJIs are reported to occur in 1.0 to 2.0% of primary and in 4.0% of revision hip and knee arthroplasties [5]. Due to increasing primary implantation rates and aging populations, the incidence of PJIs is expected to increase, which could be explained by longer prosthesis lifetime and an increase in the prevalence of co-morbidities such as obesity and diabetes [6]. PJIs can be classified as acute or chronic infections [2,7]. Acute infections are infections that present with symptoms for less than four weeks, and are further divided into early, postoperative infections (EI), and acute late, hematogenous infections (ALI). Chronic PJIs (CI) present with symptoms for more than 4 weeks [2]. Chronic PJIs can also be caused by a delayed or missed diagnosis of hematogenous PJIs [6,8].

The therapeutic management of PJIs requires a multidisciplinary approach and includes a combination of surgical and non-surgical interventions [2,5,9]. The main treatment goals are infection eradication, maintenance or improvement of joint function, and reinfection prevention. Surgical options described include debridement, antibiotics and implant retention (DAIR) with modular component exchange, and one- and two-stage prosthesis exchange [5,9]. Antibiotic treatment is an important non-surgical component in PJI treatment [10,11].

In recent years, there has been growing research and interest in the field, to establish standards for the definition, prevention, diagnosis, and treatment of PJIs following primary total hip arthroplasty (THA) and total knee arthroplasty (TKA), with the aim of improving patient outcomes and reducing the economic burden associated with revision surgeries and prolonged hospitalization [2,5,7,12,13]. The treatment of PJIs, however, remains a challenge. Relapses, in form of reinfections, are serious complications, even after appropriate PJI management. They can have a dramatic impact on a patient’s quality of life, mobility, morbidity and result in prolonged and more complex treatment [1,2]. In the current literature, there remains a paucity of evidence-based data regarding the differences of early clinical outcomes for different PJI entities. The purpose of this study, therefore, is to investigate the early outcome differences in terms of relapse-free survival, mortality, and possible complications between early, acute late, and chronic infections after primary THA and TKA.

## 2. Results

### 2.1. Sample

From 215 cases, 115 cases (111 patients) could be included in the study after applying the exclusion criteria (49.6% male; 50.4% female). Of these, 48 cases (41.7%) were PJIs after TKA and 67 (58.3%) after THA. A total of 22 cases (19.1%) were EI, 38 (33.0%) were ALI, and 55 (47.8%) were CI (Table 1).

**Figure 1:**
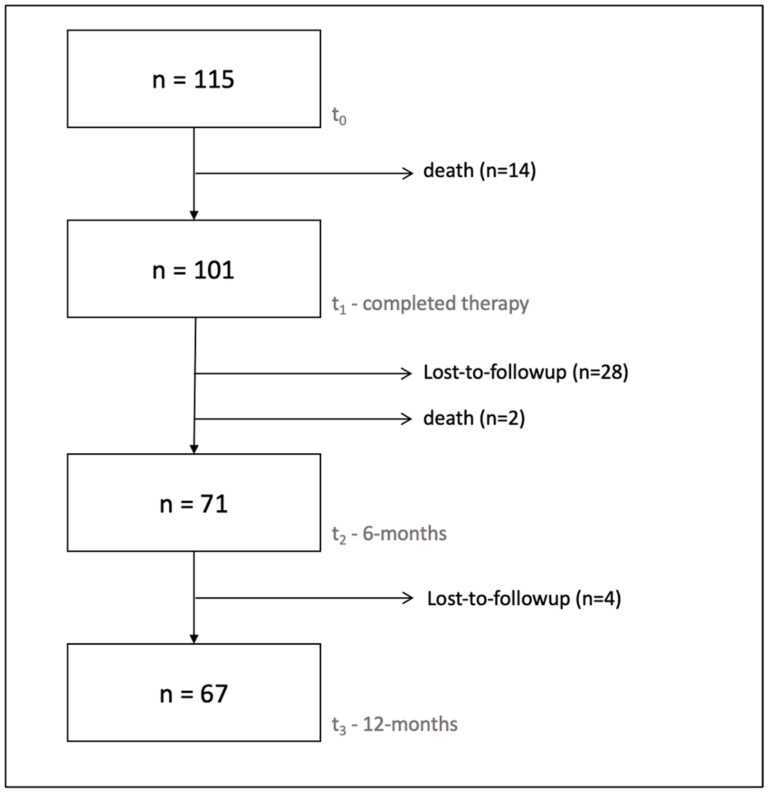
Flowchart showing the number of cases available for analysis at each study point.

Ten patients (10/111; 9.0%), representing fourteen cases (14/115; 12.2%), died before the end of treatment (t1). In total, 28 cases (28/115; 24.3%) were lost to follow-up and two patients (2/111; 1.8%) were deceased at six months after treatment completion (t2). A further four cases (4/115; 3.8%) were lost to follow-up between 6 and 12 months (t3). Overall, 71 cases (71/115; 61.7%) remained for the final evaluation at 6 months, and 67 (67/115; 58.3%) at 12 months (Figure 1).

### 2.2. Sociodemographic Characteristics, Secondary Diagnoses, and Inpatient Stay

The average age was 70.6 (±10.6) years. The average body mass index (BMI) and American Society of Anesthesiologists score (ASA score) were 30.6 (±7.8) and 2.7 (±0.6), respectively. Compared to EI and CI, patients with ALI were significantly older (*p* = 0.023; 70.1 years in EI, 74.3 years in ALI and 68.2 years in CI) and had significantly higher ASA scores (*p* = 0.031; 2.5 in EI, 2.9 in ALI and 2.6 in CI). Patients with CI were significantly younger at the time of primary implantation (*p* = 0.002; 70.1 years in EI, 64.8 years in ALI and 59.8 years in CI).

The average preoperative CRP concentration was 89.9 (±107.6) and was highest in patients with ALI (*p* = 0.011; 80.7 in EI, 136.0 in ALI and 65.6 in CI) (Table 1). There was no significant difference in the presence of comorbidities (arterial hypertension, diabetes, rheumatoid arthritis, and osteoporosis).

An average of 2.9 (±2.0) surgeries were performed per case (*p* = 0.272; 2.4 in EI, 2.6 in ALI and 3.2 in CI). The cases resulted in an average of 2.0 (±1.2) inpatient stays (*p* = 0.084; 1.4 in EI, 2.1 in ALI and 2.0 in CI) and a total of 55.1 (±39.3) inpatient days over the treatment course (*p* = 0.581; 47.5 in EI, 59.6 in ALI and 55.3 in CI). There were no significant differences between the subgroups for these endpoints.

### 2.3. Microbiology

In total, 89.0% of the patients had positive cultures at initial surgery. Of these, 74.8% were infected with a single pathogen, while 12.2% had polymicrobial infections. Patients with CI had the highest percentage of negative cultures. Patients with ALI had the highest percentage of cultures with more than one bacterium isolated (Table 2). Staphylococci were detected as the most frequent, with 66.0% of all positive cultures. Of those, 40.9% were *S. aureus*, 57.6% were coagulase-negative staphylococci (CNS), and 1.5% contained both *S. aureus* and CNS. Patients with EI had lower percentages of staphylococci (35.0%) detected than patients with ALI and CI, with 67.6% and 78.3% of the samples, respectively. Patients with EI had higher percentages of Enterobacterales, and anaerobic bacteria detected (Table 2). Blood cultures were examined in 74 of the cases, of which 34 (46.0%) were positive. Patients with ALI had a significantly higher prevalence of positive blood cultures (*p* = 0.002; 58.3% in EI, 67.9% in ALI, and 23.5% in CI). In 16 cases (21.6%), the blood cultures showed the same pathogens as the infected joint.

### 2.4. Relapse-Free Survival (RFS)

Six months after treatment completion (n = 71), 76.1% (54/71) cases were relapse free (Table 3). Reinfection was confirmed in 6.7% (1/15) of the cases with EI, compared to 28.6% in both ALI (6/21) and CI (10/35). There was no significant difference between the subgroups (*p* = 0.396). However, it should be noted that there seems to be a clear trend towards a higher RFS in patients with EI.

**Figure 2:**
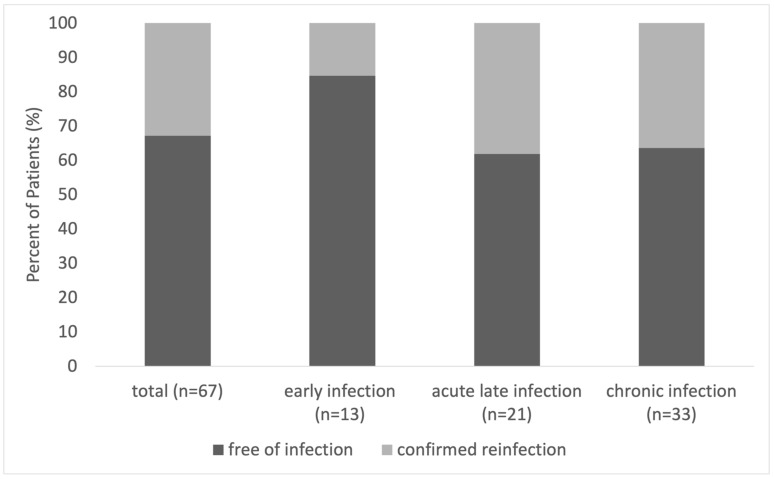
Rate of confirmed relapse-free survival 12 months after treatment completion for patients with EI, ALI, and CI.

In total, 12 months after treatment completion (n = 67), 67.1% (45/67) of the cases were relapse free. Overall, 15.4% (2/13) of patients with EI had a confirmed reinfection, compared to 38.1% (8/21) in ALI and 36.4% (12/33) in CI (Figure 2 and Table 3). There was no significant difference between the subgroups (*p* = 0.621). However, patients with EI appeared to have a higher rate of RFS. The Kaplan-Meier analysis showed no significant difference in the distribution of survival between the subgroups over the 12 months of follow-up (*p* = 0.342).

### 2.5. Mortality Analysis

**Figure 3:**
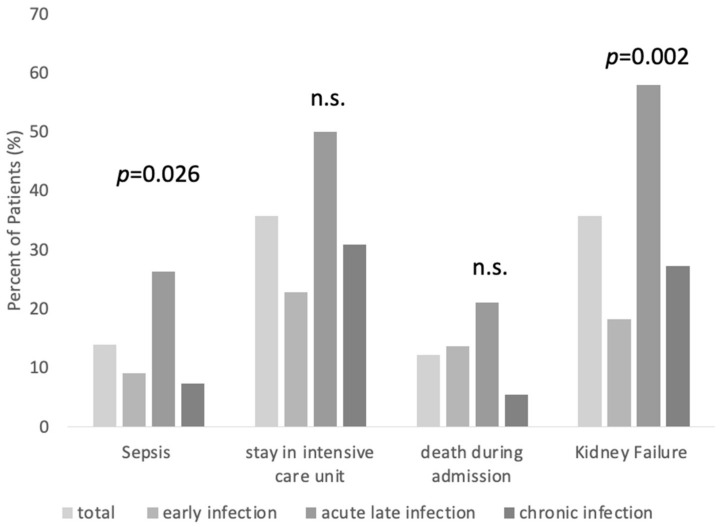
Complications within the inpatient stay for patients with EI, ALI, and CI. (n.s. = not significant, see text for explanations).

In total, 10 patients (10/111; 9.0%) (five male, five female), representing 14 (14/115; 12.2%) cases, died before treatment completion (Figure 3). On average, the patients died 9.4 days (range 0–44 days) after the last surgical intervention. Patients with ALI had a mortality rate of 21.1%, compared to 13.6% in EI and 5.5% in CI. There was no significant difference between the subgroups regarding in-house mortality (*p* = 0.076). However, it must be noted that there seems to be a trend towards higher in-house mortality in patients with ALI.

Deceased patients had a significantly higher average ASA score (*p* = 0.001, 3.2 in deceased vs. 2.6 in completed treatment), and were more likely to be admitted to the ICU (*p* ≤ 0.0001, 100.0% in deceased vs. 26.7% in completed treatment) and to be diagnosed with sepsis (*p* ≤ 0.0001; 71.4% in deceased vs. 5.9% in completed treatment). There were no significant differences in age or BMI (Table 4).

### 2.6. Complications

Overall, there were 16 (16/115; 13.9%) cases of sepsis. The average initial and maximum q-SOFA were 6.6 and 12.7, respectively. Patients with ALI were significantly more likely to be diagnosed with sepsis (*p* = 0.026, 9.1% in EI, 26.3% in ALI, and 7.3% in CI). In total, 41 (41/115; 35.7%) cases were admitted to the ICU. While 50.0% of the cases with ALI were admitted to the ICU, it were 22.7% for EI and 30.9% for CI (Figure 3). There was no significant difference in the occurrence of ICU admission between the subgroups (*p* = 0.062). Patients with ALI had a significantly higher prevalence of diagnosed kidney failure during the treatment process (*p* = 0.002, 18.18% in EI, 57.9% in ALI, and 27.3% in CI) (Figure 3).

## 3. Materials and Methods

### 3.1. Study Design

A retrospective, single-center study was conducted on all patients diagnosed with PJI between 1 January 2018 and 30 June 2019, using the ICD-code T84.5 (infection and inflammatory reaction due to internal joint prosthesis). Patient data were extracted from the hospital’s internal information system (SAP; Siemens AG Healthcare Sector, Erlangen, Germany) from May to August 2021. Written consent for the use of personal and medical data was obtained from all patients. No identifiable information about individual participants was accessible in the data analysis process. The study was approved by the ethics committee of the Medical Faculty of the University of Leipzig (approval number: 204/20-ek).

### 3.2. Endpoints

The primary endpoints of this study were mortality and the rate of relapse-free survival (RFS) at 6 and 12 months after treatment completion. Secondary endpoints were the occurrence of complications (sepsis, admission to the intensive care unit (ICU), kidney failure) and the course of the inpatient stay (number of surgeries performed, number of inpatient stays, and number of inpatient days).

### 3.3. Inclusion and Exclusion Criteria

Patients were eligible to the study if they:

(i) Underwent THA or TKA.
(ii) Had PJI according to the definition of the Musculoskeletal Infection Society criteria for periprosthetic joint infections. [14].

Patients were excluded if they:

(i) Had a prosthesis of a joint other than the hip or the knee or if they had a hemi-prothesis.
(ii) Had a course of treatment that started before 1 January 2018, and after 30 June 2019.
(iii) Had aseptic loosening.
(iv) Had a periprosthetic fracture.
(v) Had a post-traumatic primary endoprosthesis implantation.

In case of radiographic and/or clinical suspicion of aseptic loosening, the patients received pre-operative and intraoperative examinations for infection exclusion. It was therefore clearly verified when loosing was due to aseptic loosening and not an infection, and these patients were excluded. Periprosthetic fractures and post-traumatic endoprosthesis implantations were excluded, as current literature suggests that those have a different course of disease and different susceptibility to infections, which influences their treatment [15,16,17,18,19,20].

### 3.4. Diagnostic and Treatment Process

The institutional standard operating procedure (SOP) for the clinical management of PJIs was applied, which included a standardized diagnostic (laboratory examinations, imaging, and screening for secondary foci), and a surgical and antibiotic treatment regimen for all patients (Supplementary Figures S1–S3). Each patient’s treatment was discussed every 14 days by an interdisciplinary panel consisting of orthopedic surgeons, microbiologists, infectious disease specialists, and pharmacists.

The treatment algorithm differentiated between early infections (EI), acute late infections (ALI), and chronic infections (CI), and was adapted from proposed definitions and classifications as described in the literature [2,7,21]. Symptoms persisting for less than 4 weeks were classified as acute infections, while symptoms extending beyond 4 weeks were classified as chronic infections (CI). Acute infections that occurred within four weeks of primary implantation were classified as early infections (EI), while those occurring later than four weeks since primary implantation were classified as acute late infections (ALI) [2]. If the patients had an infection in more than one joint, each joint was viewed as a separate case (Supplementary Figure S1).

Both EI and ALI were treated as acute infections with debridement, antibiotics, and implant retention (DAIR) and modular component exchange under the calculated administration of antibiotics. When the pathogen was resistant against the used antibiotics, subsequent lavage was performed in a second surgery under a resistance-based antibiotic treatment. These patients received a maximum of two intermittent lavages. If still persistent, further procedures were performed according to the treatment algorithm for chronic infections (Supplementary Figure S2).

Patients with chronic infections received a complete change of the prosthesis (one-stage change in patients without significant comorbidities like diabetes mellitus, rheumatoid arthritis, peripheral arterial occlusive disease, etc.; two-stage change in patients with significant comorbidities or presence of fistula). Temporary implanted spacers were loaded with gentamycin. In case of infection persistence, a maximum of two intermittent lavages under resistance-based antibiotic treatment were performed. Prosthesis reimplantation was carried out after 6 to 24 weeks. Salvage procedures included Girdlestone arthroplasty, knee arthrodesis or a permanent fistula (Supplementary Figure S3).

All patients received antibiogram-adjusted intravenous antibiotics for 14 days post-surgery. A short intravenous antibiotic duration was chosen as several studies at that time suggested that long-term systemic (oral or intravenous) antibiotic treatment was not superior to a short treatment interval and even showed higher rates of antibiotic-related severe adverse events [22,23,24,25].

### 3.5. Follow-Up

After complete treatment, all patients received appointments for follow-up examinations, which included clinical and radiological evaluations. The patients were classified as lost-to-follow-up if they did not attend the follow-up appointments and could not be contacted by telephone.

The outcome was assessed at 6 and 12 months after treatment completion and infection eradication. Successful PJI eradication was defined according to the Delphi criteria [26]. Infection relapse was defined as the presence of clinical and laboratory signs of inflammation requiring revision surgery and/or antibiotic treatment. Relapse-free survival was defined as the absence of clinical and laboratory signs of inflammation and the absence of further revision surgery or antibiotic treatment. No distinction was made between early and delayed relapses.

### 3.6. Data Analysis

Statistical data analysis was performed using GraphPad Prism 8.4.1 (GraphPad Software, Inc., San Diego, CA, USA), Microsoft Excel and IBM SPSS, version 24.0 (SPSS Inc., IBM Corp., Armonk, NY, USA). Categorical data were presented as frequencies (n) and percentages (%), and continuous data were described as means and standard deviations (SD). Subgroup analysis was performed for EI, ALI, and CI. To assess the differences between groups, a chi-squared test was used for categorical data or one-way analysis of variance (ANOVA-test) for continuous data. The level of statistical significance was set at a two-sided *p*-value of <0.050. The probability of relapse-free survival was estimated using a Kaplan-Meier analysis. Subgroups were compared using the log-rank test. An alpha-level of <0.050 was considered significant.

## 4. Discussion

The aim of this retrospective, single-center study was to investigate the early-outcome differences between early, late acute, and chronic PJIs in terms of relapse-free survival, mortality, and possible complications. Identifying certain outcome differences and risk factors during the early treatment course of different PJI entities could be beneficial to optimize future diagnostic and treatment strategies. This study has shown that trends in the early outcomes and occurrence of complications between EI, ALI, and CI can be identified.

This study found no statistically significant difference in RFS between EI, ALI, and CI. However, there appeared to be a clear trend towards better outcomes in patients with EI, while patients with ALI and CI had comparable rates of confirmed reinfection. These results are in accordance with the literature. Wouthuyzen-Bakker et al. found statistically lower treatment success rates with DAIR in patients with ALI, compared to patients with EI, when infection was caused by *Staphylococcus* spp. over a total follow-up-period of 2 years (46.0% success in ALI vs. 76.0% in EI) [27]. Both Chang et al. and Vilchez et al. have also found that treatment success was higher in postsurgical (acute) PJI compared to hematogenous PJI [28,29]. Similarly, other studies have reported high relapse rates in patients with ALI, ranging from 21.0% up to 55.0% [30,31,32,33,34,35,36]. On the other hand, Renz et al. reported higher RFS rates for hematogenous PJIs (79% at 12 months, 69.0% at 24 months and 62.0% at 48 months) [31]. Konigsberg et al. reported recurrent infection requiring surgery in 21.0% of their patients at a mean of 56 months (range of 25–124 months) [33]. Sigmund et al. showed that all complications requiring revision in two-stage arthroplasty occurred within 2 years and that 17.2% of the patients had a persistent infection in the endoprosthesis-free interval. Overall, 10.0% were revised because of an infection after a mean period of 7.8 months (range of 0.3–22.6 months) [37]. In a recent multicenter study, which included 340 patients with late hematogenous PJI from 27 centers, a failure rate of 45.0% was found. The greatest predictor for successful treatment was the exchange of mobile parts during DAIR. Significant independent preoperative risk factors were age over 80 years and C-reactive protein >150 mg/L [34]. Ultimately, the failure rates of this multicenter study are comparable to the presented results and emphasize the challenges in the treatment of ALI. In the presented single-center study, additional insights on the cooccurrence of early post-operative complications (sepsis, admission to ICU, kidney failure, mortality) could be demonstrated. These are particularly high in patients with ALI. The presented results support the conclusion of Wouthuyzen-Bakker et al., that ALI may require different treatment algorithms [34].

However, it is important to note that the above results should all be compared with caution, as the follow-up periods, study set-ups and definitions for relapse vary greatly between the studies. Some studies only specify mean follow-up periods ranging from 0.2 months to 4.9 years [30,31,32,33,35,36]. 

This study also found no significant difference in the mortality rates of the subgroups. The overall in-house mortality was 9.0%. However, it should be noted that there was a trend towards higher in-house mortality rates in patients with ALI. Deceased patients had a higher health burden, had significantly higher average ASA scores, a higher prevalence of sepsis and a higher rate of ICU admission compared to patients with complete treatment. Similarly, Drain et al. have shown that mortality during PJI treatment was associated with pre-surgical morbidity and frailty, but not the treatment [38]. Renz et al. have found similar results, describing death in 11.0% of their cohort, of which 5.0% could be directly attributed to hematogenous PJI [31]. Other studies evaluating one-year mortality after PJI treatment have described mortality rates ranging from 2.5% to 10.6% [39,40,41,42,43].

The large heterogeneity in current literature on RFS and mortality may also reflect the still existing discrepancies and inconsistencies in the clinical research in the field of PJI, e.g., the definition of failure and success criteria, and inconsistent and incomparable treatment strategies. This impedes conclusive comparisons between studies.

There also appeared to be a trend towards higher ICU admission rates in patients with ALI. The overall mortality in the ICU was 34.0% (n = 14/41). Pöll et al. also found a high mortality rate in patients with PJI admitted to the ICU (21.0%). Independent factors associated with a worse outcome were renal replacement therapy and a higher Simplified Acute Physiology Score II (SAPSII) and Charlson Comorbidity Index (CCI) on admission [44]. Maloum et al. reported a mortality rate of 20.0%. Factors associated with a worse outcome were diabetes mellitus, an ASA score above three, and acute infections [45].

Finally, compared to patients with EI and CI, patients with ALI were significantly older at admission and had a significantly higher mean ASA score. They also had significantly higher preoperative CRP levels and were more likely to develop sepsis or kidney failure during treatment. The presented results suggest that patients with ALI tend to have worse outcomes and a higher risk of systematic complications. This could be because hematogenous PJI has been described to be diagnosed later and to have more virulent pathogens with more resistances [46]. The above parameters may indicate that patients with ALI have a higher disease burden, which should be considered in the treatment. However, the results may be partly explained by the higher average age of the patients at admission. According to the authors, kidney failure may contribute especially to worse outcomes during the treatment process. Future research is needed to determine whether early strategies to improve kidney function can lead to better outcomes. Overall, the presented results suggest that patients with ALI should be monitored more frequently to prevent relapse and to respond as quickly as possible to signs of reinfection. This could be achieved, for example, by arranging more frequent follow-up appointments that include clinical and laboratory examinations.

Furthermore, this study shows that there are differences in the microbiological colonization between different PJI entities. Within the described cohort, a significant proportion, 74.8%, manifested colonization with a single bacterium. In 12.2% of the cases, there were polymicrobial infections, which were highest in patients with ALI. Patients with CI had the highest percentage of negative cultures. These highlight potential challenges in culturing pathogens in case of CI for diagnostic purposes, possibly due to biofilm formation or prior antimicrobial treatment as described in other studies [47,48]. The presented results suggest that there might be a potential shift in microbial composition associated with the progression of joint infections. Patients with EI had a lower occurrence of staphylococci and higher proportions of Enterobacterales and anaerobic bacteria. These findings are in accordance with the current literature [49,50] and underscore the importance of considering the infection stage in the selection of antimicrobial therapy to ensure effective treatment. The antibiotic treatment also plays an important role in infection eradication. This study used a short interval of antibiotic treatment for all patients, as studies at the time of this study’s conception suggested that long-term systemic (oral or intravenous) antibiotic treatment is non-superior to a short treatment regimen [22,23,24,25]. Considering the current literature, the authors clearly recommend antibiotic treatment intervals longer than 14 days, as described in this study. However, it must also be critically noted that there is still no conclusive evidence that long treatment intervals of 12 weeks and more are associated with better outcomes than shorter intervals (e.g., 6 weeks) [11,51,52]. Furthermore long treatment intervals of antibiotics can be associated with an increased occurrence of adverse events [53]. The optimal duration for the administration of systemic antibiotics remains to be defined [54].

This study has certain limitations. First, this study was retrospective and focused on a single center. The authors, however, justify this approach as a standardized diagnostic and treatment algorithm has been used for each subgroup according to current clinical practice. In addition, only a small study cohort was analyzed, no power analysis was performed, and the subgroups varied greatly in size. Furthermore, the follow-up period was relatively short, consisting of 12 months. This follow-up was chosen and justified by the authors as the early clinical outcomes were the focus of this study to define which patients are at risk for relapse and complications. As shown in the results, patients with ALI should be observed and evaluated at shorter intervals. In addition to that, other authors reported that most relapses occur within the first 12 months after treatment completion [37,55]. Finally, it must be noted that there are different definitions for periprosthetic infections.

## 5. Conclusions

In conclusion, there are considerable differences in patient characteristics and outcomes between different PJI entities. Patients with EI seem to have better outcomes, whereas patients with ALI tend to have a higher incidence of kidney failure and sepsis, and higher relapse and mortality rates, although no statistically significant difference in RFS or mortality was found between EI, ALI, and CI. Further prospective multicenter studies with longer follow-up periods should be performed to validate the presented results and to develop adjusted interdisciplinary treatment protocols for the management of different PJI entities.

## Figures and Tables

**Table 1:** Sociodemographic characteristics of the cases (* = significant *p*-value; SD = standard deviation; see text for explanations).

	Total (n = 115)		Early Infection (EI) (n = 22)		Acute Late Infection (ALI) (n = 38)		Chronic Infection (CI) (n = 55)		
	Absolute	%	Absolute	%	Absolute	%	Absolute	%	
Female	58	50.4	12	54.5	19	50.0	27	49.1	
Male	57	49.6	10	45.5	19	50.0	28	50.9	
Total hip replacement	67	58.3	17	77.3	17	44.7	33	60.0	
Total knee replacement	48	41.7	5	22.7	21	55.3	22	40.0	
	Average	SD	Average	SD	Average	SD	Average	SD	*p*-value
BMI	30.6	7.8	30.3	9.2	31.4	7.0	30.3	7.9	0.768
Age at admission	70.6	10.6	70.1	8.7	74.3	8.7	68.2	11.8	0.023 *
Age at initial implantation	63.5	12.1	70.1	8.7	64.8	10.7	59.8	13.1	0.002 *
ASA score	2.7	0.6	2.5	0.5	2.9	0.6	2.6	0.6	0.031 *
CRP	89.9	107.6	80.7	73.8	136.0	122.8	65.6	102.5	0.011 *

**Table 2:** Microbiological pathogen detection of material obtained at initial surgery.

	Total (n = 115)		Early Infection (EI) (n = 22)		Acute Late Infection (ALI) (n = 38)		Chronic Infection (CI) (n = 55)	
	Absolute	%	Absolute	%	Absolute	%	Absolute	%
Negative microbiological culture	15	13.0	2	9.1	4	10.5	9	16.4
Positive microbiological culture	100	87.0	20	90.9	34	89.5	46	83.6
Multiple pathogens in culture	14	14.0	2	1.0	6	17.6	7	15.2
Staphylococci *	66	57.4	7	31.8	23	60.5	36	65.5
Enterococci	10	8.7	2	9.1	4	10.5	4	7.3
Enterobacterales	21	18.3	7	31.8	8	21.1	6	10.9
Anaerobic bacteria	10	8.7	4	18.2	2	5.3	4	7.3
Corynebacteria	2	1.7	1	4.5	0	0.0	1	1.8
Streptococci	7	6.1	1	4.5	3	7.9	3	5.5
Spore formers	4	3.5	0	0.0	2	5.3	2	3.6
Yeast-like fungi	1	0.9	0	0.0	1	2.6	0	0.0
* *S. aureus*	27	40.9	4	57.1	11	47.8	12	33.3
Coagulase-negative staphylococci (CNS)	38	57.6	3	42.9	12	52.2	23	63.9
*S. aureus* and CNS	1	1.5	0	0.0	0	0.0	1	2.8

* Division of Staphylococci into *S. aureus* and coagulase negative staphylococci. Percentages are calculated from the total number of Staphylococci.

**Table 3:** Relapse-free survival and rate of reinfection for total cases and the subgroups.

		Total (n = 71)		EI (n = 15)		ALI (n = 21)		CI (n = 35)		
		Absolute Number	%	Absolute Number	%	Absolute Number	%	Absolute Number	%	*p*-Value
After 6 months	Free of infection	54	76.1	14	93.3	15	71.4	25	71.4	0.396
	Reinfection	17	23.9	1	6.7	6	28.6	10	28.6	
		Total (n = 67)		EI (n = 13)		ALI (n = 21)		CI (n = 33)		
After 12 months	Free of infection	45	67.2	11	84.6	13	61.9	21	63.6	0.621
	Reinfection	22	32.8	2	15.4	8	38.1	12	36.4	

**Table 4:** Comparison between patients deceased before therapy completion and patients with complete treatment (* = significant *p*-value; SD = standard deviation; see text for explanations).

	Death before Therapy Completion (n = 14)		Complete Treatment (n = 101)		
	Absolute	%	Absolute	%	
Female	7	50.0	50	49.5	
Male	7	50.0	51	50.5	
Total hip replacement	9	64.3	58	57.4	
Total knee replacement	5	35.7	43	42.6	
	Average	SD	Average	SD	*p*-value
Age at admission	75.0	9.1	70.0	10.7	0.096
BMI	31.2	7.3	30.6	7.9	0.790
ASA score	3.2	0.8	2.6	0.5	0.001 *
Stay in intensive care unit	14	100.0	27	26.7	0.000 *
Sepsis	10	71.4	6	5.9	0.000 *

## Data Availability

The data presented in this study are available from the corresponding author on reasonable request due to privacy and ethical restrictions.

## Supplementary Materials

The following supporting information can be downloaded at: https://www.mdpi.com/article/10.3390/antibiotics13030198/s1, Figure S1. Internal algorithm for screening for PJI. Figure S2. Internal treatment algorithm for the treatment of acute PJI. Figure S3. Internal treatment algorithm for the treatment of chronic PJI.

## Abbreviations

ALI	acute late infection
CI	chronic infection
EI	early infection
RFS	relapse free survival
ICU	intensive care unit
PJI	periprosthetic joint infection
THA	total hip arthroplasty
TKA	total knee arthroplasty
