# Supplementary material for: Early-Outcome Differences between Acute and Chronic Periprosthetic Joint Infections—A Retrospective Single-Center Study
Source: Antibiotics (Basel). 2024 Feb 20;13(3):198. doi: 10.3390/antibiotics13030198 (PMC10967401; doi:10.3390/antibiotics13030198)
Supplement: Supplementary file 1 [file antibiotics-13-00198-s001.zip › S1_fig.pdf]

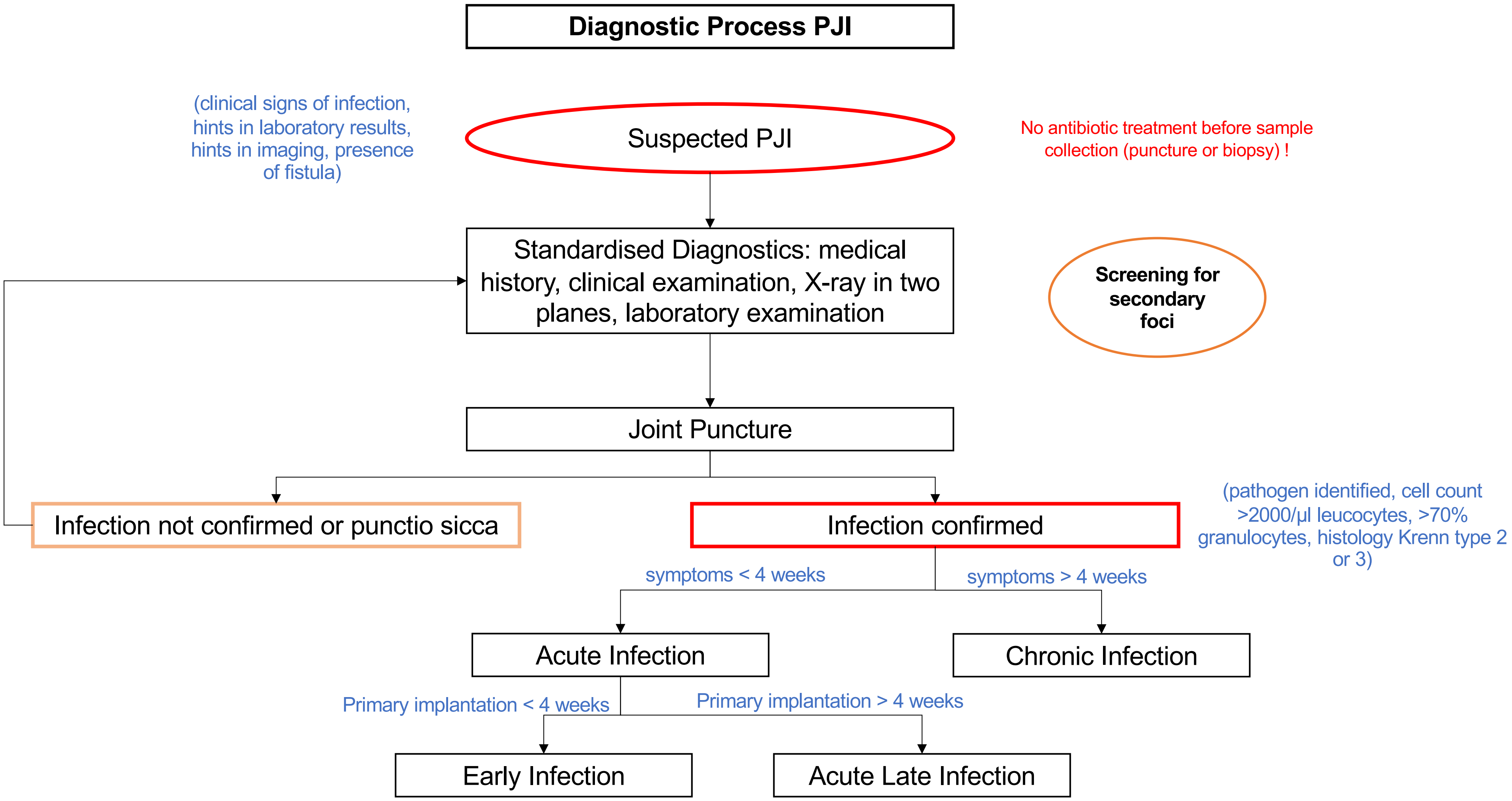
Diagnostic Process PJI
(clinical signs of infection, hints in laboratory results, hints in imaging, presence of fistula)
Suspected PJI
No antibiotic treatment before sample collection (puncture or biopsy) !
Standardised Diagnostics: medical history, clinical examination, X-ray in two planes, laboratory examination
Screening for secondary foci
Joint Puncture
Infection not confirmed or punctio sicca
Infection confirmed
(pathogen identified, cell count >2000/µl leucocytes, >70% granulocytes, histology Krenn type 2 or 3)
symptoms < 4 weeks
symptoms > 4 weeks
Acute Infection
Chronic Infection
Primary implantation < 4 weeks
Primary implantation > 4 weeks
Early Infection
Acute Late Infection
