# Supplementary figures and images for: Early-Outcome Differences between Acute and Chronic Periprosthetic Joint Infections—A Retrospective Single-Center Study
Source: Antibiotics (Basel). 2024 Feb 20;13(3):198. doi: 10.3390/antibiotics13030198 (PMC10967401; doi:10.3390/antibiotics13030198)

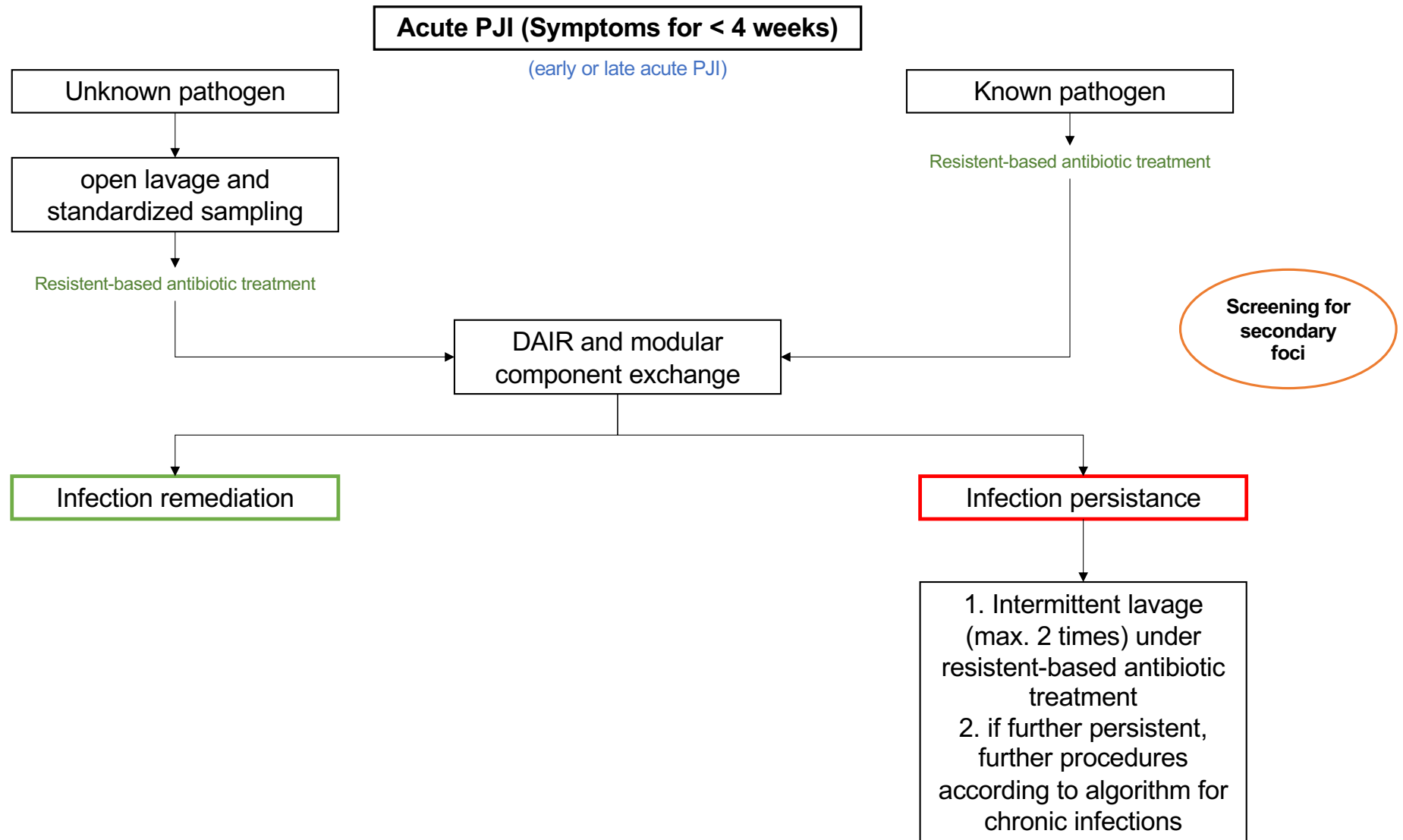

Supplement: Supplementary file 1 [file antibiotics-13-00198-s001.zip › S2_fig.pdf]

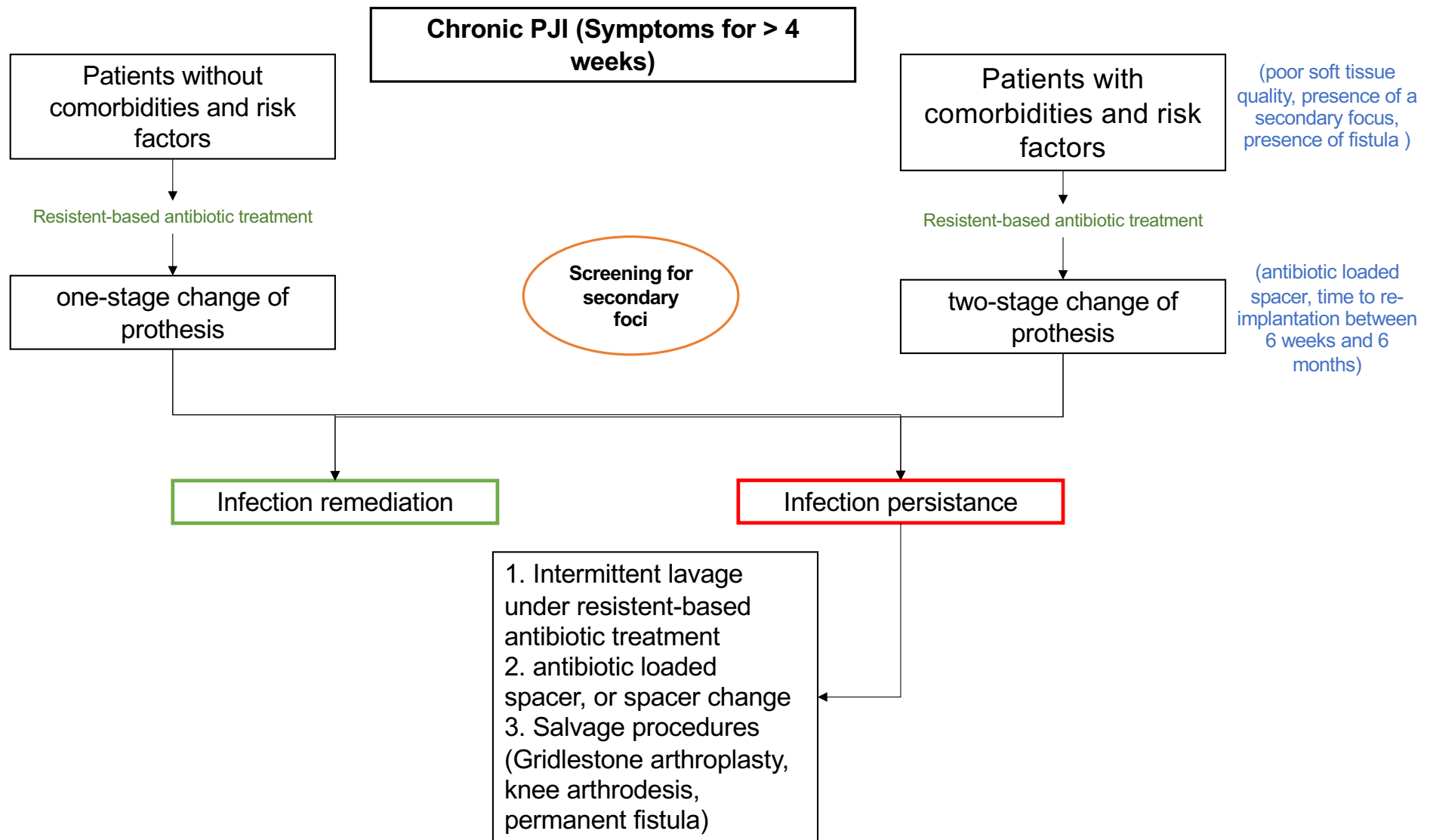

Supplement: Supplementary file 1 [file antibiotics-13-00198-s001.zip › S3_fig.pdf]
